# Supplementary material for: Empirical methods for controlling false positives and estimating confidence in ChIP-Seq peaks
Source: BMC Bioinformatics. 2008 Dec 5;9:523. doi: 10.1186/1471-2105-9-523 (PMC2628906; doi:10.1186/1471-2105-9-523)
Supplement: Additional File 1 — A variety of html documents from the USeq web site detailing the available applications, their best usage, output file type descriptions, command line menus, etc. [file 1471-2105-9-523-S1.zip › USeqUserGuides/JavaDoc.zip!/JavaDoc/bioroot/plasmid/PlasmidSpreadSheet.html]

PlasmidSpreadSheet


|  |  |  |
| --- | --- | --- |
| **Overview** \| **Package** \| **Class** \| **Use** \| **Tree** \| **Deprecated** \| **Index** \| **Help** | |  |
| **PREV CLASS**   **NEXT CLASS** | **FRAMES**    **NO FRAMES**     **All Classes** |  |
| SUMMARY: NESTED \| FIELD \| CONSTR \| METHOD | DETAIL: FIELD \| CONSTR \| METHOD |  |


---


## bioroot.plasmid Class PlasmidSpreadSheet

```
java.lang.Object
  javax.servlet.GenericServlet
      javax.servlet.http.HttpServlet
          bioroot.plasmid.PlasmidSpreadSheet
```

**All Implemented Interfaces:**: java.io.Serializable, javax.servlet.Servlet, javax.servlet.ServletConfig

---

``` public class PlasmidSpreadSheet extends javax.servlet.http.HttpServlet ```

**Author:**
:   nix

**See Also:**: Serialized Form

---

**Constructor Summary**

`PlasmidSpreadSheet()`


| **Method Summary** | |
| --- | --- |
| `void` | `doGet(javax.servlet.http.HttpServletRequest request, javax.servlet.http.HttpServletResponse response)` |
| `void` | `doPost(javax.servlet.http.HttpServletRequest request, javax.servlet.http.HttpServletResponse response)` |
| `static PlasmidBean[]` | `fetchPlasmidBeans(java.lang.String sql, DBUtil bioRoot)`             Build PlasmidBean[] from Plasmid table in database. |
| `static java.lang.String` | `fetchPlasmidTableRows(UserBean userBean, PlasmidBean[] beans, int[] startStop, DBUtil bioRoot, boolean checked, boolean allTabs)`             Returns table rows for every oligo according to a Preference, if allTabs==true, then all features are returned in a tab delimited format, otherwise its HTML. |
| `static void` | `makeAndSetPlasmidBeans(javax.servlet.http.HttpSession session, DBUtil bioRoot, UserBean userBean)`             Makes an array of PlasmidBeans based on Users PlasmidPreferences and sets them in the session. |
| `static PlasmidBean[]` | `orderPlasmids(java.lang.String featureName, UserBean userBean, PlasmidBean[] beans, DBUtil bioRoot)`             Orders plasmids, sort of nasty, can't figure another way, reflection is way too slow. |

**Methods inherited from class javax.servlet.http.HttpServlet**

`service`

**Methods inherited from class javax.servlet.GenericServlet**

`destroy, getInitParameter, getInitParameterNames, getServletConfig, getServletContext, getServletInfo, getServletName, init, init, log, log`

**Methods inherited from class java.lang.Object**

`equals, getClass, hashCode, notify, notifyAll, toString, wait, wait, wait`

**Constructor Detail**

### PlasmidSpreadSheet

```
public PlasmidSpreadSheet()
```


**Method Detail**

### doGet

```
public void doGet(javax.servlet.http.HttpServletRequest request,
                  javax.servlet.http.HttpServletResponse response)
           throws javax.servlet.ServletException,
                  java.io.IOException
```

:   **Overrides:**: `doGet` in class `javax.servlet.http.HttpServlet`
:   **Throws:**: `javax.servlet.ServletException`: `java.io.IOException`

---


### doPost

```
public void doPost(javax.servlet.http.HttpServletRequest request,
                   javax.servlet.http.HttpServletResponse response)
            throws javax.servlet.ServletException,
                   java.io.IOException
```

:   **Overrides:**: `doPost` in class `javax.servlet.http.HttpServlet`
:   **Throws:**: `javax.servlet.ServletException`: `java.io.IOException`

---


### makeAndSetPlasmidBeans

```
public static void makeAndSetPlasmidBeans(javax.servlet.http.HttpSession session,
                                          DBUtil bioRoot,
                                          UserBean userBean)
```

:   Makes an array of PlasmidBeans based on Users PlasmidPreferences and sets them in the session.

---


### fetchPlasmidTableRows

```
public static java.lang.String fetchPlasmidTableRows(UserBean userBean,
                                                     PlasmidBean[] beans,
                                                     int[] startStop,
                                                     DBUtil bioRoot,
                                                     boolean checked,
                                                     boolean allTabs)
```

:   Returns table rows for every oligo according to a Preference, if allTabs==true, then all features are
    returned in a tab delimited format, otherwise its HTML. Set startStop to 0,0 if you want everything.

---


### fetchPlasmidBeans

```
public static PlasmidBean[] fetchPlasmidBeans(java.lang.String sql,
                                              DBUtil bioRoot)
```

:   Build PlasmidBean[] from Plasmid table in database.

---


### orderPlasmids

```
public static PlasmidBean[] orderPlasmids(java.lang.String featureName,
                                          UserBean userBean,
                                          PlasmidBean[] beans,
                                          DBUtil bioRoot)
```

:   Orders plasmids, sort of nasty, can't figure another way, reflection is way too slow.


---


|  |  |  |
| --- | --- | --- |
| **Overview** \| **Package** \| **Class** \| **Use** \| **Tree** \| **Deprecated** \| **Index** \| **Help** | |  |
| **PREV CLASS**   **NEXT CLASS** | **FRAMES**    **NO FRAMES**     **All Classes** |  |
| SUMMARY: NESTED \| FIELD \| CONSTR \| METHOD | DETAIL: FIELD \| CONSTR \| METHOD |  |


---
